# Supplementary material for: Identification of the occurrence and potential mechanisms of heterotopic ossification associated with 17-beta-estradiol targeting MKX by bioinformatics analysis and cellular experiments
Source: PeerJ. 2022 Jan 3;10:e12696. doi: 10.7717/peerj.12696 (PMC8734462; doi:10.7717/peerj.12696)
Supplement: Supplemental Information 1 [file peerj-10-12696-s001.docx]

| Supplementary table 1. Primer sequences | |  |  |
| --- | --- | --- | --- |
|  | Forward and reverse | Length | Tm |
| RUNX2 | F: 5' TGGTTACTGTCATGGCGGGTA 3' | 21 | 62.9℃ |
|  | R: 5' TCTCAGATCGTTGAACCTTGCTA 5' | 23 | 61.1℃ |
| GAPDH | F: 5' GGAGCGAGATCCCTCCAAAAT 3' | 21 | 61.6℃ |
|  | R: 5' GGCTGTTGTCATACTTCTCATGG 5' | 23 | 60.9℃ |
