# Supplementary figures and images for: Identification of the occurrence and potential mechanisms of heterotopic ossification associated with 17-beta-estradiol targeting MKX by bioinformatics analysis and cellular experiments
Source: PeerJ. 2022 Jan 3;10:e12696. doi: 10.7717/peerj.12696 (PMC8734462; doi:10.7717/peerj.12696)

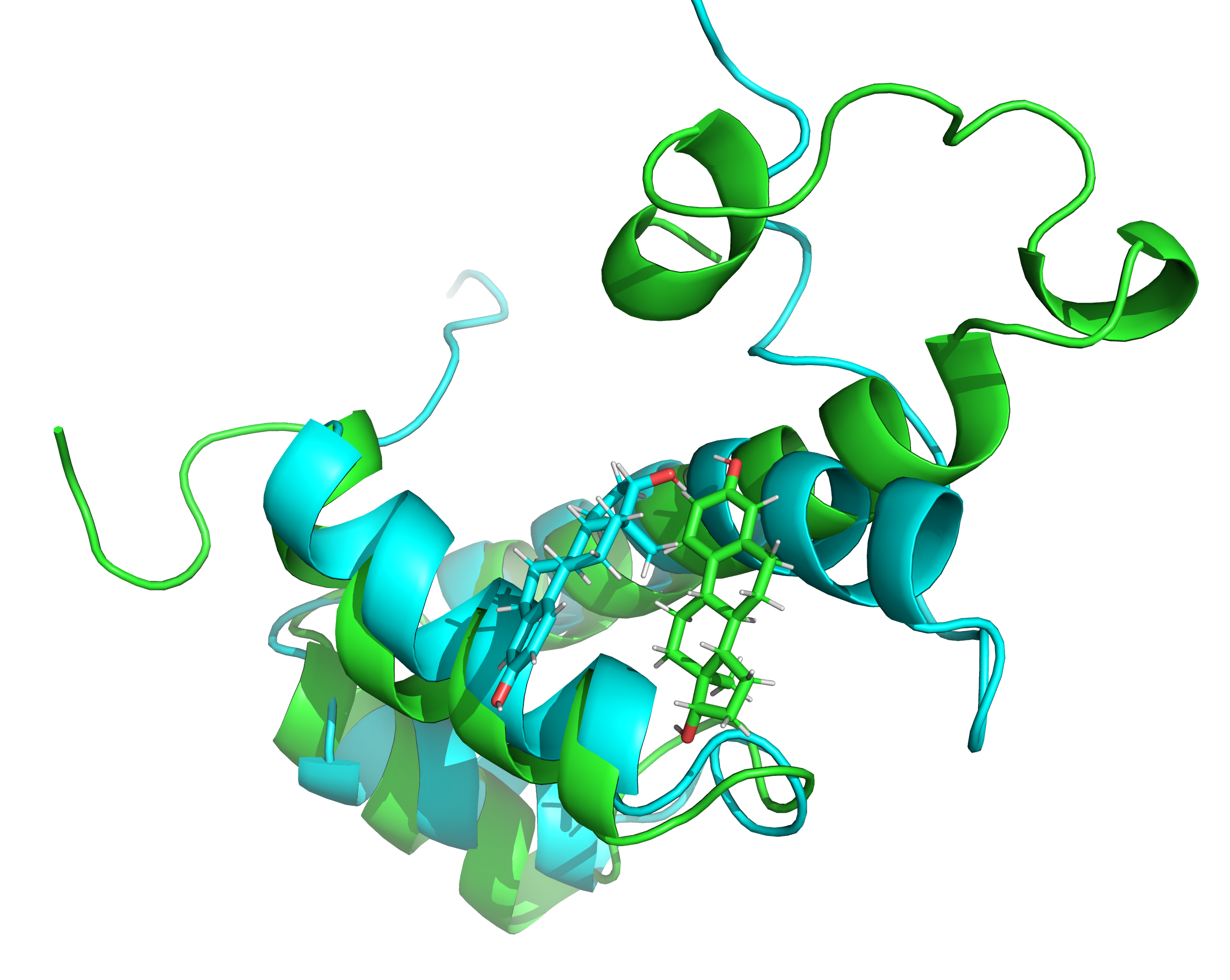

Supplement: Supplemental Information 4 [file peerj-10-12696-s004.zip › MD raw data/c1/pose.png]

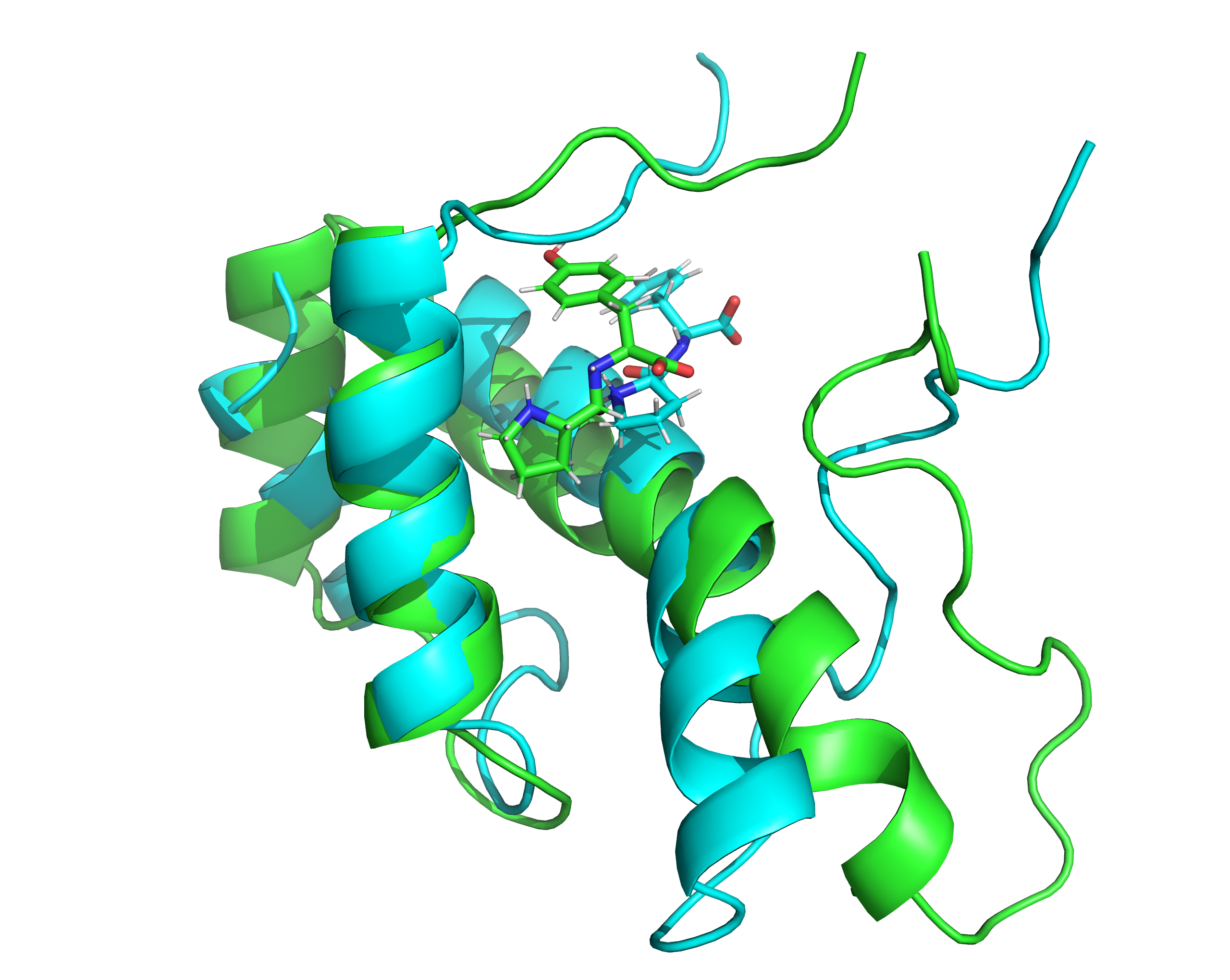

Supplement: Supplemental Information 4 [file peerj-10-12696-s004.zip › MD raw data/p1/pose2.png]
